# PAK1 Is Involved in the Spindle Assembly during the First Meiotic Division in Porcine Oocytes

**DOI:** 10.3390/ijms24021123

**Published:** 2023-01-06

**Authors:** Lei Peng, Yijing He, Weihan Wang, Yajie Chu, Qixin Lin, Rong Rui, Qiao Li, Shiqiang Ju

**Affiliations:** MOE Joint International Research Laboratory of Animal Health and Food Safety, College of Veterinary Medicine, Nanjing Agricultural University, Nanjing 210095, China

**Keywords:** pig, oocytes, meiosis, PAK1, spindle

## Abstract

P21-activated kinase 1 (PAK1), as a member of the PAK family, has been implicated in various functions during somatic mitosis; however, less is known about its role during oocyte meiosis. Herein, we highlight the indispensable role of PAK1 in regulating spindle assembly and cell cycle progression during the first meiotic division of porcine oocytes. First, we found that the activated PAK1 expressed dynamically, and its subcellular localization was tightly associated with the spindle dynamics during meiosis in porcine oocytes. Specific inhibition of PAK1 activity by inhibitor targeting PAK1 activation-3 (IPA-3) led to impaired extrusion of the first polar body (PB1); with most of the IPA-3-treated oocytes arrested at germinal vesicle breakdown (GVBD) and subjected to failure of bipolar spindle formation. However, the adverse effects caused by IPA-3 on oocytes could be restored by reducing disulfide bonds between PAK1 and IPA-3 with dithiothreitol (DTT) treatment. Furthermore, the co-immunoprecipitation assay revealed that PAK1 interacted directly with Aurora A and transforming acidic coiled coil 3 (TACC3), providing an additional explanation for the similar localization of Aurora A and activated PAK1. Additionally, inhibiting the activity of PAK1 decreased the expression of p-Aurora A and p-TACC3; however, the reduced activity of Aurora A and TACC3 could be restored by DTT. In conclusion, PAK1 plays a crucial role in the proper assembly of the spindle during the first meiotic division of porcine oocytes, and the regulation of PAK1 is associated with its effects on p-Aurora A and p-TACC3 expression.

## 1. Introduction

The spindle, a microtubule-based structure, is responsible for the proper division of all eukaryotic cells that accomplish equal segregation of genetic material [1]. During mitosis, cell division control protein 42 (Cdc42) is the main Rho GTPase that contributes to spindle orientation, centrosome integrity, and bi-orientation of chromosomes attached to microtubules [2]. As a main downstream effector of the Cdc42 GTPase, p21-activated kinase 1 (PAK1) has also been implicated in various functions to do with regulating microtubule dynamics during mitosis [3]. Being a member of the PAK family, PAK1 is an evolutionarily conserved family of serine/threonine kinases identified initially in budding yeast and designated Ste20 [4], which regulates cell proliferation, cell migration, and cytoskeletal dynamics [5]. PAK1 is activated by Cdc42 or Rac binding to the p21-binding domain (PBD) [6]. In mitosis, activated PAK1 is crucial for the accumulation of γ-tubulin/γ-TuRCs and the nucleation of interphase microtubules through its association with G-protein-coupled receptor-interacting protein 1 (GIT1) or PAK-interacting exchange factor-beta (β-PIX) [7]. As the cell reaches metaphase, GIT1/β-PIX recruits PAK1 to the centrosomes, where it is activated, and then phosphorylates Aurora A at threonine 288 to support centrosome dynamics and chromosome alignment [8]. Moreover, inhibiting the kinase activity of PAK1 by inhibitor targeting PAK1 activation-3 (IPA-3), an allosteric inhibitor of PAK1 kinase activity, leads to hyperacetylated microtubules and a lack of microtubule network integrity [9].

The meiosis of mammalian oocytes involves two successive cell divisions without an intermediate replicative phase, characterized by accurate segregation of the maternal genome and highly asymmetric cytoplasmic partitioning [10]. Moreover, mammal oocytes differ from somatic cells in the organization of spindle microtubules, since there are no centrosomes in meiosis; and this asymmetric division is controlled by microtubules and microfilament cytoskeletons, referred to as centriole-free strategies for assembling spindle [11,12]. Any errors in these processes can result in aneuploidy and embryonic development defects.

Although multiple functions of PAK1 have been implicated in mitosis, little information is available on the roles of PAK1 in meiosis. It has been reported that suppression of PAK1 leads to the disorganized spindle as well as failure of chromatin condensation in mouse oocytes [13,14]. Nevertheless, a distinction is noted between porcine and mouse oocytes in how spindle formation occurs. In mouse oocytes, the nucleation of microtubules (MTs) relies on the function of acentrioloar microtubule-organizing centers (aMTOCs) [15], and the depletion of pericentrin (PCNT) from the germinal vesicle breakdown (GVBD) stage, an aMTOCs material, results in disorganized spindle [16]. However, in porcine oocytes, the aMTOCs are absent before meiosis I, and the nucleation of microtubules in the oocytes is initially mediated by chromatin and Ran GTP gradients [17]. It is unclear whether PAK1 is required for the assembly of microtubules in porcine oocytes, and its function during the meiotic maturation of porcine oocytes remain unknown. In this study, the possible role of PAK1 was investigated, and the potential mechanism was also explored in the meiosis of porcine oocytes.

## 2. Results

### 2.1. Dynamic Distribution and Expression of p-PAK1 during Meiotic Maturation in Porcine Oocytes

**Figure 1:**
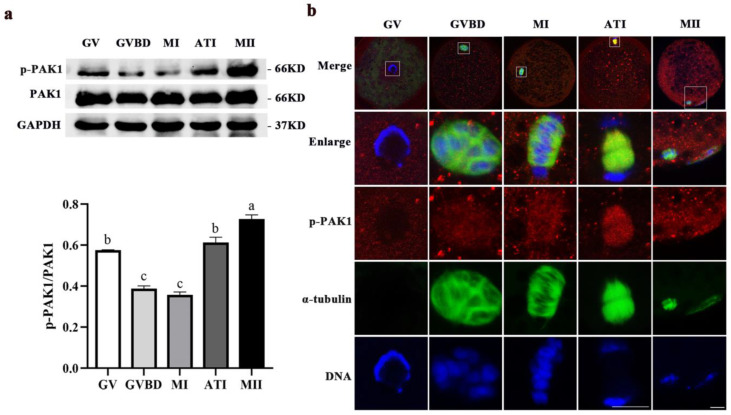
Localization and relative expression of PAK1 meiotic maturation in porcine oocytes. (**a**) The relative expression of p-PAK1 and total PAK1 in porcine oocytes undergoing meiosis. ^a, b, c^ Values with different superscript letters above columns indicate significant differences (*p* < 0.05). (**b**) Subcellular localization of p-PAK1 during meiosis in porcine oocytes (*n* = 40). Red, p-PAK1. Green, α-tubulin. Blue, chromosome. Scale bars are 10 μm. GV, germinal vesicle; GVBD, germinal vesicle breakdown; MI, metaphase I; ATI, anaphase-telophase I; MII, metaphase II.

As shown in Figure 1a, p-PAK1 was dynamically expressed at all meiotic stages in porcine oocytes and showed higher protein levels in the metaphase II (MII) stage. Figure 1b showed that when the oocytes were at the germinal vesicle (GV) stage, the ring-like chromatin was in the cytoplasm and the α-tubulin was scattered outside the germinal vesicle; meanwhile, p-PAK1 was observed to disperse in the cytoplasm. When the oocytes reached the GVBD stage, the chromatin condensed into chromosomes and a network-like α-tubulin gathered around the chromosomes. At the metaphase I (MI) stage, barrel-like bipolar spindles were formed, and the chromosomes were arranged at the metaphase plate of the spindle. At the anaphase-telophase (ATI) stage, the homologous chromosomes segregated, and the α-tubulin was distributed between the two sets of segregated chromosomes. At the same time, p-PAK1 was visualized surrounding α-tubulin and then appeared to enrich at the spindle region from the GVBD to ATI stages. When oocytes progressed to the MII stage, the first polar body (PB1) was extruded; and the α-tubulin was assembled into a MI meiotic spindle and scattered at the PB1. Moreover, p-PAK1 was distributed near the spindle in the cytoplasm of oocyte and the polar body. This subcellular localization pattern suggested that PAK1 may be correlated with the meiotic spindle dynamics during porcine oocyte maturation.

### 2.2. Inhibition of PAK1 Activity Affected Porcine Oocyte Maturation

**Figure 2:**
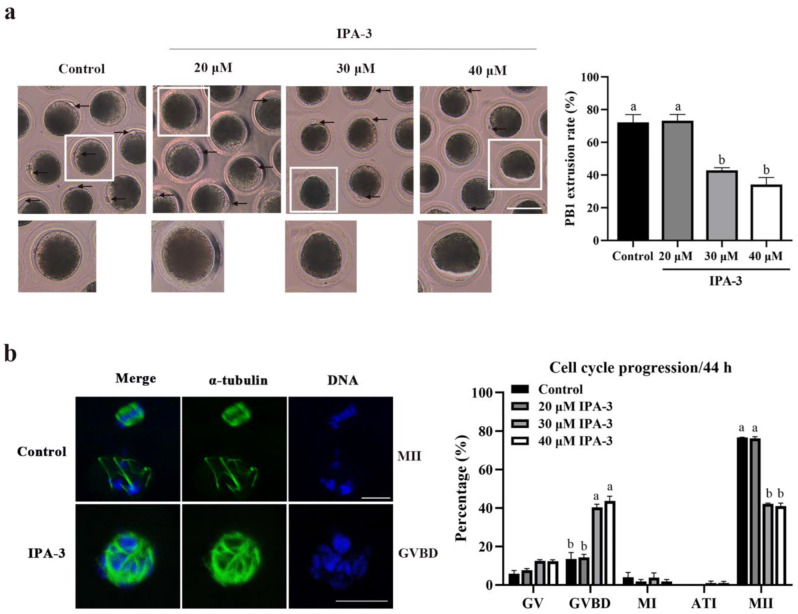
Effects of IPA-3 treatment on maturation and cell cycle progression of porcine oocytes. (**a**) Representative images of PB1 extrusion and PB1 extrusion rate in control and IPA-3 treated groups after 44 h of culture (*n* = 104 for the control group; *n* = 105 for 20 µM IPA-3 group; *n* = 105 for 30 µM IPA-3 group; *n* = 105 for 40 µM IPA-3 group). Arrow, the PB1. Scale bars are 100 μm. (**b**) PAK1 inhibition disrupted the cell cycle progression of porcine oocytes (*n* = 103 for the control group; *n* = 105 for 20 µM IPA-3 group; *n* = 104 for 30 µM IPA-3 group; *n* = 105 for 40 µM IPA-3 group). Green, α-tubulin. Blue, chromosome. Scale bars are 10 μm. ^a, b^ Values with different superscript letters above columns indicate significant differences (*p* < 0.05). PB1, the first polar body; GVBD, germinal vesicle breakdown; MII, metaphase II.

To determine the possible roles of PAK1 during meiotic maturation, the oocytes were cultured in the presence of various concentrations of IPA-3 (0, 20, 30 and 40 μM) for 44 h, and then the PB1 extrusion and the meiotic progression of the oocytes were examined. As shown in Figure 2a, most of the oocytes in the control group extruded the PB1 and reached the MII stage, while only a small percentage of the IPA-3 treated oocytes extruded the PB1 as the concentration of inhibitor increased. Compared with the control group (72.18 ± 4.89%), the rate of PB1 extrusion was significantly decreased, to 42.86 ± 1.65% (*p* < 0.05) and 34.28 ± 4.29% (*p* < 0.05) when treated with 30 and 40 μM concentration of IPA-3, respectively; and there was no significant difference when treated with 20 μM IPA-3 (73.33 ± 3.81%, *p* > 0.05). Additionally, as presented in Figure 2b, higher proportions of the oocytes were arrested at the GVBD stage after 30 μM (40.41 ± 1.65%, *p* < 0.05) and 40 μM (43.80 ± 2.39%, *p* < 0.05) IPA-3 treatment than that in the control group (13.53 ± 3.34%). Based on these results, a 30 μM IPA-3 concentration was used for further exploration.

### 2.3. Inhibition of PAK1 Activity Resulted in Disorganized Spindles in Porcine Oocytes

**Figure 3:**
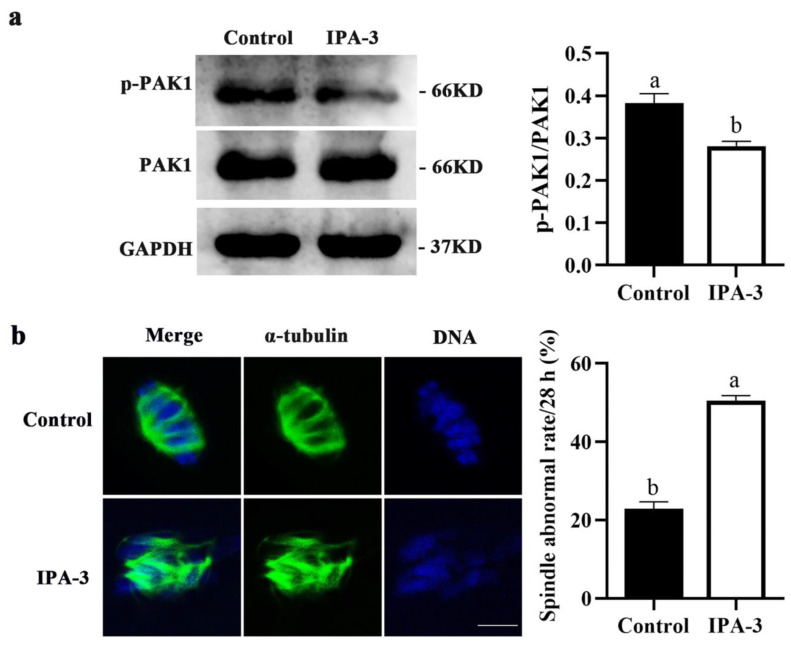
Effects of IPA-3 treatment on spindle assembly during the first meiotic division in porcine oocytes. (**a**) Effect of IPA-3 treatment on the expression of p-PAK1 after 28 h of culture. (**b**) Effect of PAK1 inhibition on the spindle morphology and chromosome alignment in porcine oocytes (*n* = 100 for the control group; *n* = 101 for 30 µM IPA-3 group). Green, α-tubulin. Blue, chromosome. Scale bars are 10 μm. ^a, b^ Values with different superscript letters above columns indicate significant differences (*p* < 0.05).

To further investigate why oocytes failed to progress to the MI stage after IPA-3 treatment, the PAK1 activity and the subcellular structure of the spindles in the oocytes were also examined. As shown in Figure 3a, the expression of p-PAK1 (*p* < 0.05) was markedly decreased after 30 μM IPA-3 treatment for 28 h, and most IPA-3-treated oocytes displayed disorganized spindles with multiple asters and misaligned chromosomes. The percentage of the PAK1-inhibited oocytes with abnormal spindles was significantly higher than that in the control group (50.47 ± 1.30% vs. 22.96 ± 1.75%, *p* < 0.05, Figure 3b). Together, the above data demonstrated that PAK1-inhibited oocytes were arrested at the GVBD stage and unable to progress to the MI stage, due to failure of bipolar spindle assembly during the first meiotic division.

### 2.4. Dithiothreitol(DTT) Restored Oocytes Maturation and Normal Spindle Formation

**Figure 4:**
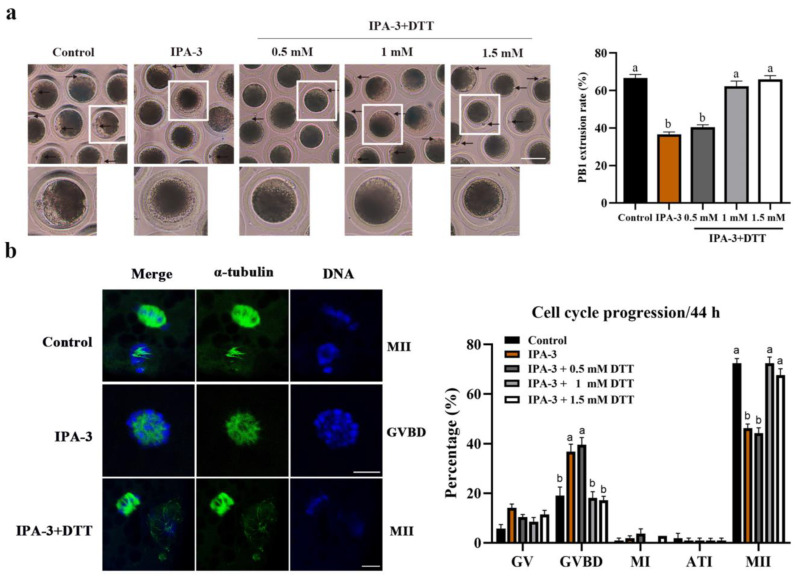
Effects of DTT on the maturation and cell cycle progression of IPA-3-treated oocytes. (**a**) Representative images of PB1 extrusion and the rate of PB1 extrusion in the control, IPA-3 and IPA-3 + DTT groups after 44 h of culture in vitro (*n* = 105 for control group; *n* = 104 for 30 µM IPA-3 group; *n* = 104 for IPA-3 + 0.5 mM DTT group; *n* = 106 for IPA-3 + 1 mM DTT group; *n* = 106 for IPA-3 + 1.5 mM DTT group). Arrow, the PB1. Scale bars are 100 μm. (**b**) DTT restored the cell cycle progression of IPA-3-treated oocytes (*n* = 105 for control group; *n* = 106 for 30 µM IPA-3 group; *n* = 106 for IPA-3 + 0.5 mM DTT group; *n* = 105 for IPA-3 + 1 mM DTT group; *n* = 105 for IPA-3 + 1.5 mM DTT group). Green, α-tubulin. Blue, chromosome. Scale bars are 10 μm. ^a, b^ Values with different superscript letters above columns indicate significant differences (*p* < 0.05). PB1, the first polar body; GVBD, germinal vesicle breakdown; MI, metaphase I; MII, metaphase II.

To further confirm the role of PAK1 in porcine oocytes during meiosis, DTT, a reduction against the disulfide bond between PAK1 and IPA-3, was used to restore the PAK1-inhibited oocytes [18]. As presented in Figure 4a, few oocytes treated with IPA-3 extruded the PB1 and reached the MII stage; but most of the IPA-3 treated oocytes successfully extruded the PB1 after being treated with DTT and cultured for 44 h, which is nearly the same as the control group. Compared with the IPA-3 group (36.51 ± 1.33%, *p* < 0.05), the PB1 extrusion rate of the PAK1-inhibited oocytes significantly increased to 62.22 ± 2.77% and 66.01 ± 1.91%, respectively, when treated with 1 and 1.5 mM DTT, and showed no significant difference compared with the control group (66.67 ± 1.90%, *p* > 0.05). Moreover, after treatment with 1 mM DTT, the proportion of PAK1-inhibited oocytes arrested at the GVBD stage had decreased significantly more than that in the IPA-3 treatment group (18.10 ± 2.52% vs. 36.83 ± 3.03%, *p* < 0.05, Figure 4b).

**Figure 5:**
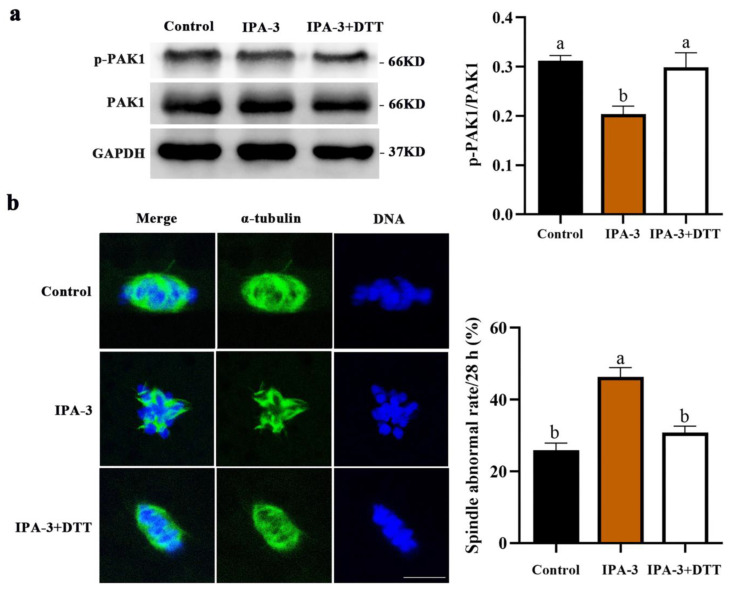
Effect of DTT on spindle assembly during the first meiotic division in IPA-3-treated oocytes. (**a**) Effect of DTT on the expression of p-PAK1 in the IPA-3-treated oocytes after 28 h of culture in vitro. (**b**) Effect of DTT on the spindle morphology and chromosome alignment in the IPA-3-treated oocytes (*n* = 108 for the control group; *n* = 110 for 30 µM IPA-3 group; *n* = 107 for the IPA-3 + 1 mM DTT group). Green, α-tubulin. Blue, chromosome. Scale bars are 10 μm. ^a, b^ Values with different superscript letters above columns indicate significant differences (*p* < 0.05).

According to the given results, a concentration of 1 mM DTT was used to restore the inhibition of IPA-3 on PAK1 activity. After being cultured for 28 h, compared with the IPA-3 treatment group, the expression of p-PAK1 was increased (*p* < 0.05, Figure 5a) in the IPA-3 + DTT group. And as shown in Figure 5b, the abnormality rate of spindle in the IPA-3 + DTT group had also decreased significantly more than that in the IPA-3 treatment group (30.86 ± 1.74% vs. 46.35 ± 2.53%, *p* < 0.05). Thus, the meiotic defects of the IPA-3-treated oocytes could be reversed by DTT, suggesting that PAK1 was indispensable in spindle assembly during the first meiotic division of porcine oocytes.

### 2.5. Inhibition of PAK1 Decreased the Activity of Aurora A and TACC3

**Figure 6:**
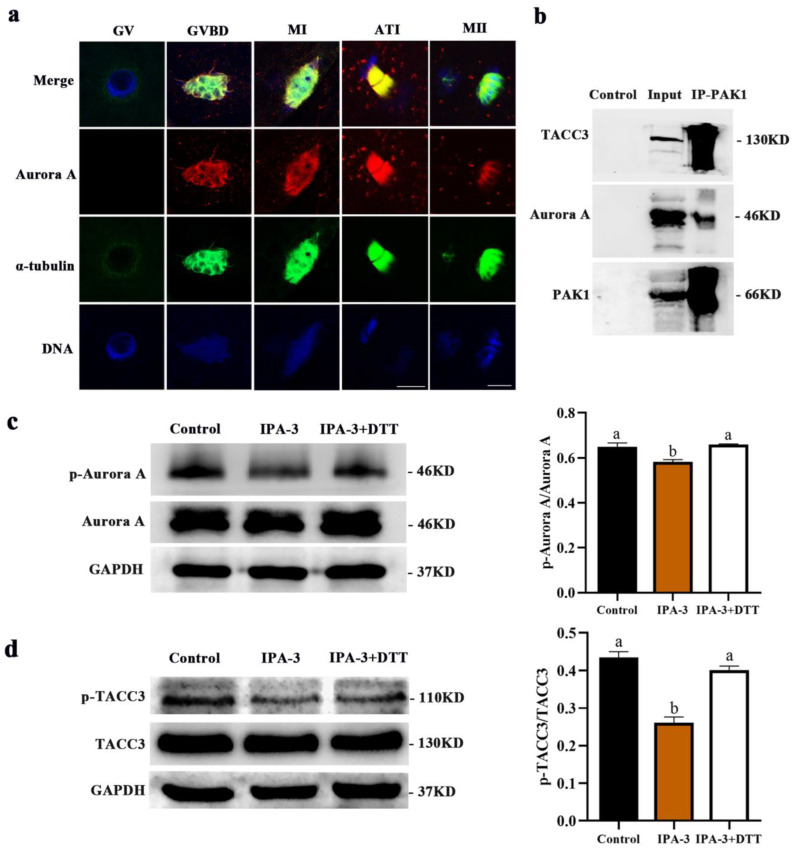
Effect of PAK1 inhibition on the expression of p-Aurora A and p-TACC3 during the first meiotic division (28 h). (**a**) Subcellular localization of Aurora A during meiosis in porcine oocytes (*n* = 40). Red, Aurora A. Green, α-tubulin. Blue, chromosome. Scale bars are 10 μm. (**b**) Co-immunoprecipitation results indicated that PAK1 was interacted with Aurora A and TACC3. (**c**) The expression of p-Aurora A in control, 30 µM IPA-3 and IPA-3 + 1 mM DTT groups. (**d**) The expression of p-TACC3 in control, 30 µM IPA-3 and IPA-3 + 1 mM DTT groups. ^a, b^ Values with different superscript letters above columns indicate significant differences (*p* < 0.05). GV, germinal vesicle; GVBD, germinal vesicle breakdown; MI, metaphase I; ATI, anaphase-telophase I; MII, metaphase II.

As shown in Figure 6a, Aurora A was enriched at the spindle region from the GVBD stage and all subsequent meiotic stages. And the subcellular localization of Aurora A was closely correlated to the dynamic distribution of α-tubulin, which was similar to the localization pattern of p-PAK1. The co-immunoprecipitation result further confirmed that PAK1 interacted directly with Aurora A and transforming acidic coiled coil 3 (TACC3) during the first meiotic division (Figure 6b). Moreover, PAK1 inhibition by IPA-3 led to the decreased expression of p-Aurora A and p-TACC3 at the MI stage, as revealed by immunoblotting (Figure 6c,d, *p* < 0.05). However, the decreased expression of p-Aurora A and p-TACC3 could be restored by DTT (Figure 6c,d, *p* < 0.05).

## 3. Discussion

Though PAK1 has been implicated in various mitotic functions during mitosis in somatic cells [19,20], little information is available about the meiotic roles of PAK1 during meiosis in mammalian oocytes. In the present study, the possible roles of PAK1 in the porcine oocyte during meiosis were addressed, and the results reveal both that PAK1 is essential for spindle assembly during the first meiotic maturation and that this regulation might be related to its effect on the activity of Aurora A and TACC3.

In porcine oocytes, activated PAK1 was found to be dynamically expressed during meiotic divisions and mainly distributed over the cytoplasm at the GV stage, followed by a distinct accumulation at the α-tubulin from GVBD to MII stage. The subcellular localization pattern of the activated PAK1 is similar to that of some microtubule-associated proteins, such as MTR120 [21] and TPX2 [22]. In mitosis, PAK1 has been reported to accumulate at the centrosomes and spindles and regulate astral microtubules during metaphase [23], as well as phosphorylate tubulin cofactor B (TCoB) to regulate microtubules dynamics [24]. Given the evidence above, it is inferred that PAK1 may be involved in regulating the meiotic division of porcine oocytes and plays a potential role relevant to the spindle dynamics. 

IPA-3 is an allosteric inhibitor of PAK1 through the mechanism of non-ATP competition and binding with regulatory domains of PAK1 in covalent ways, thus allowing for analyzing the function of PAK1 [25,26]. A previous study showed that IPA-3 was found to significantly inhibit the activation of PAK1, even in the presence of 1 mM concentrations of ATP [27]; and 20 to 40 μM IPA-3 treatment significantly inhibited the growth of hepatocellular carcinoma (HCC) cells cultured in vitro, through suppressing the activation of PAK1 and subcellular translocation of nuclear factor light-chain enhancer of activated B cells (NF-κB) [28]. In human hematopoietic cells, 20 μM IPA-3 treatment markedly induced cell death and affected cell adhesion to fibronectin as the activity of PAK1 was suppressed [29]. In this study, after 30 μM IPA-3 treatment, the activated PAK1 level was markedly decreased and the PB1 extrusion rate of the oocytes was significantly decreased, with most of the oocytes being arrested at the GVBD stage. During the GVBD-to-MI transition process in oocytes, the precise assembly and dynamic distribution of spindle microtubules are critical for correct MI spindle formation and chromosome alignment [30]. Most of the PAK1-inhibited oocytes exhibited extreme spindle aberrance with the chromosome misalignment, suggesting that abnormal assembly of spindle during metaphase I may be responsible for the cell cycle arrest in the PAK1-inhibited oocytes in this study. These results revealed that PAK1 plays a crucial role in the spindle assembly of porcine oocytes during the first meiotic division. 

The mechanism of inhibiting activation of PAK1 rests on the disulfide bond of IPA-3, which can be reversed by DTT, a reducing agent, and reduces PAK1 inhibition by IPA-3 [18,31]. In the *Xenopus* oocyte cytoplasmic extract system, the amount of bounding between PAK1 and IPA-3 was decreased significantly after treatment with 0.1 to 20 mM DTT for 13 min [25]. Thus, DTT can further verify the function of PAK1 in porcine oocytes during the first meiotic division. In this study, 1 mM DTT did restore the decreased expression of activated PAK1 inhibited by IPA-3. In addition, after the addition of DTT to the PAK1-inhibited oocytes, spindle formation in the first metaphase returned to its normal work, which resulted in the percentage of PB1 extrusion in IPA-3 treated oocytes being significantly increased, and the proportion of oocytes arrested at the GVBD stage sharply decreasing. Based on these results, it was confirmed that activated PAK1 is indispensable for the organization of microtubules in pig oocytes during the first meiotic division. 

Female meiosis is characterized by a pair of asymmetrically positioned meiotic spindles that drag chromosomes into tiny and undeveloped polar bodies; and it is the aMTOCs that are responsible for the spindle pole organization [32,33]. Moreover, during the assembly of meiotic spindles, the nucleation activity of microtubules is regulated by Ran GTP [34]. Both microtubule dynamics and the proper construction of spindles are dependent on the regulation of various protein-protein interactions; prominent among which being the interaction of Aurora A with TACC3. TACC3 was shown to regulate microtubule polymerization by Aurora A-dependent phosphorylation [35,36]. Mutations of the critical Aurora A targeting site, serine 558, in TACC3 led to the loss of astral microtubules and disruption of the localization of γ-tubulin ring complex (γ-TuRC) proteins at the spindle pole [37]. Several proteins have been reported to interact with Aurora A and TACC3 to regulate the spindle dynamics, such as NDEL1 [38], PLK1 [39] and AIBP [40]. 

The interaction between Aurora A and PAK1 has been previously demonstrated in mitosis [8]. Activated PAK1 binds to Aurora A and phosphorylates it at threonine 288, maintaining centrosome dynamics as a result [41]. Nevertheless, this interaction is less known in meiosis and deserves further investigation. Next, we tried to explore the potential mechanism of PAK1 on meiotic spindle dynamics. The localization data suggested that the subcellular localization between Aurora A and PAK1 is similar in porcine oocytes during meiosis, and Co-IP assay showed that PAK1 interacts directly with Aurora A and TACC3. Moreover, the inhibition of PAK1 activity by IPA-3 led to the activity inhibition of both Aurora A and TACC3. These results demonstrated that PAK1 affects the spindle dynamic by interacting with Aurora A and TACC3 during the first meiotic division in porcine oocytes.

## 4. Materials and Methods

### 4.1. Antibodies and Chemicals

The rabbit polyclonal ani-PAK1 pSer204 antibody, anti-PAK1 antibody, anti-Aurora A pThr288 antibody, and anti-TACC3 pSer558 antibody were purchased from Signalway Antibody (College Park, MD, USA). Mouse monoclonal anti-Aurora A antibody was acquired from Abcam (Cambridge, UK). Rabbit polyclonal anti-GAPDH antibody was procured from Proteintech Group (Chicago, IL, USA). Rabbit monoclonal anti-TACC3 antibody, TRITC-labeled goat anti-rabbit IgG and Cyc3-labeled goat anti-mouse IgG were obtained from HuaAn Biotechnology (Zhejiang, China). Mouse monoclonal anti-α-tubulin-FITC antibody and Hoechst 33342 were purchased from Sigma (St. Louis, MO, USA). HRP-labeled goat anti-rabbit IgG and HRP-labeled goat anti-mouse IgG were supplied from Beyotime Biotechnology (Shanghai, China). IPA-3 was purchased from Selleck Chemicals (Houston, TX, USA). All other chemicals and reagents used in this study were purchased from Sigma-Aldrich (St. Louis, MO, USA), unless otherwise specified.

### 4.2. Collection and In Vitro Maturation of Oocytes

All procedures with animals have been conducted in accordance with the guidelines of the Institutional Animal Care and Use Committee of Nanjing Agricultural University (Approval number: IACUC2019055), China. The ovaries were obtained from prepubertal gilts at a local slaughterhouse, then were transported to the laboratory in sterile saline solution 0.9% (*w*/*v*) within 2 h. The cumulus oocyte complexes (COCs) with multiple layers of intact cumulus cells and homogeneous cytoplasm were selected by aspiration from 3 to 6-mm diameter follicles and transferred to pre-equilibrated TCM199 medium (Gibco BRL, Gaithersburg, MD, USA), containing 0.1% (*w*/*v*) polyvinyl alcohol (PVA), 3.05 mM D-glucose, 0.91 mM sodium pyruvate, 0.57 mM cysteine, 10 ng/mL epidermal growth factor (EGF), 10 IU/mL PMSG and hCG (Ningbo Hormonal Reagents Co., Ltd., Zhejiang, China), 10% (*v*/*v*) porcine follicular fluid (pFF), 7.5 mg/mL penicillin, and 5.0 mg/mL streptomycin, and then cultured at 38.5 °C in 5% CO_2_ with saturated humidity, as reported previously [42]. According to the experimental design, oocytes were collected after being cultured for 0, 22, 28, 36 and 44 h, when most oocytes were supposed to GV, GVBD, MI, ATI and MII respectively [43], allowing for the following research.

### 4.3. Experimental Design

#### 4.3.1. Effects of PAK1 Inhibition on the Meiotic Division of Porcine Oocytes

Initially, the protein expression and subcellular localization of PAK1 and p-PAK1 were examined in porcine oocytes at different meiotic stages (GV, GVBD, MI, ATI and MII). Next, the IPA-3―a specific inhibitor of PAK1 activity [44]―was dissolved in dimethylsulfoxide (DMSO) and diluted with TCM199 culture medium to the final concentrations of 0, 20, 30 and 40 μM to treat the oocytes during in vitro maturation. After 44 h of culture, a total of 104, 105, 105 and 105 oocytes from each group (0, 20, 30 and 40 μM) were collected to evaluate the PB1 extrusion; and 103, 105, 104 and 105 oocytes in each group (0, 20, 30 and 40 μM) were collected to analyze the percentages of oocytes arrested at the GV, GVBD, MI, ATI, and MII stages. Based on the results of meiotic progression analysis above, a total of 100 oocytes from each group (0 and 30 μM) were collected for immunoblotting to measure p-PAK1 expression; and 100 and 101 oocytes from each group (0 and 30 μM) were collected for immunofluorescence staining to assess the subcellular structure of spindles after 28 h of culture, when most oocytes were supposed to reach the MI stage. 

#### 4.3.2. Effects of DTT on the Meiotic Division of IPA-3 Inhibited Porcine Oocytes

DTT is a reduction of the disulfide bond between PAK1 and IPA-3 [18]. Based on the above results (that IPA-3 treatment caused oocytes to be arrested at the GVBD), the IPA-3 (30 μM) treated oocytes were collected after 22 h of culture, washed for three times, and then incubated with different concentrations of DTT (0.5, 1 and 1.5 mM) for 13 min [25], and finally transferred into normal TCM199 medium for the latter oocyte cultures. The oocytes in normal TCM199 medium were used as the control group; the oocytes treated with 30 μM IPA-3 were the IPA-3 group; and the oocytes initially treated with 30 μM IPA-3 and then restored with DTT (0.5, 1 and 1.5 mM) were the IPA-3 + DTT group. After a total of 44 h culture, a total of 105, 104, 104, 106 and 106 oocytes from the control, IPA-3 and DTT groups (0.5, 1 and 1.5 mM) were collected for examination of PB1 extrusion; and a total of 105, 106, 106, 105 and 105 oocytes from each group were collected for the evaluation of meiotic progression. Based on the results of meiotic progression analysis above, a total of 100 oocytes from the control, IPA-3, and IPA-3 + DTT (1 mM) groups were collected, respectively, for immunoblotting to measure p-PAK1 expression; and 108, 110, and 107 oocytes from each group were collected for immunofluorescence staining to assess the subcellular structure of spindles after 28 h of culture. Finally, the expression levels of Aurora A, activated Aurora A, TACC3, and activated TACC3 were further examined in the control, IPA-3, and IPA-3 + DTT (1 mM) group by immunoblotting

### 4.4. Immunofluorescence Staining

The oocytes were gathered and fixed in 4% paraformaldehyde at room temperature (RT) for 30 min and then transferred into permeabilization solution (1% Triton X-100 in PBS) at RT for 8 h. After incubation for 1 h in PBS containing 1% BSA, the oocytes were incubated with primary antibodies (1:200) overnight at 4 °C. Having been washed three times in a washing buffer (0.1% Tween 20 in PBS), the oocytes were then incubated with secondary antibodies (1:200) and anti-α-tubulin antibody labeled with FITC (1:200) at 37 °C for 2 h separately. Next, the oocytes were stained with Hoechst 33342 (10 μg/mL in PBS) for 10 min for DNA counterstaining. Finally, the oocytes were mounted on glass slides and examined using a laser scanning microscope (Zeiss LSM700 META, Oberkochen, Germany).

### 4.5. Immunoblotting

The oocytes (approximately 100 for each group) were lysed in 15 µL ice-cold Laemmli buffer and heated at 95 °C for 10 min, then stored at −20 °C for immunoblotting. Cell lysates with equal protein were separated by 10% SDS-PAGE and transferred into polyvinylidine fluoride (PVDF) membranes (Millipore, Billerica, MA, USA), using semi-dry transfer methods. Following that, the membranes were blocked with 5% nonfat dried milk at 37 °C for 2 h and subsequently incubated with the primary antibodies (1:1000) at 4 °C overnight. They were then incubated with the secondary antibodies conjugated with horseradish peroxidase (HRP) (1:1000) at 37 °C for 2 h. After washing, the membrane was visualized with enhanced chemiluminescence (ECL) solution (Biosharp, Hefei, China) and analyzed by comparing its signal intensity to that of GAPDH, using the Image J 1.8.0 (National Institutes of Health, USA).

### 4.6. Co-Immunoprecipitation

Co-immunoprecipitation (Co-IP) was conducted using the IP/Co-IP kit (Absin, Shanghai, China) according to the manufacturer’s instructions. In short, approximately 700 oocytes were added to the lysis buffer (500 μL) containing 1 mM phenylmethanesulfonyl fluoride (PMSF) (Absin, Shanghai, China) for 1 h at 4 °C. Next, the cell lysate was incubated with anti-PAK1 antibody (3 μL) at 4 °C overnight. Following this, protein A and G beads (5 μL) were used to treat the lysates at 4 °C for 3 h. The immunoprecipitate was then resuspended with a 1×SDS loading buffer (20 μL), followed by immunoblotting. Based on the previous report [45], the IP-PAK1 group was the cell lysate incubated with rabbits polyclonal anti-PAK1 antibody. Additionally, the input group was the cell lysate without anti-PAK1 antibody, and the control group was the cell lysate incubated with rabbit IgG antibody.

### 4.7. Statistical Analysis

Statistical analysis was performed with SPSS (Statistics Production for Service Solution, Version 22.0). Three replicates were conducted for each experiment. The difference between groups was analyzed by one-way ANOVA, followed by Duncan’s multiple comparisons tests. The results are presented as means ± standard error. A probability (P) below 0.05 was considered significant.

## 5. Conclusions

In summary, the results of the present study suggested that PAK1 is crucial for proper spindle assembly during meiotic maturation of porcine oocytes. The regulatory role of PAK1 may be mediated by its interaction with Aurora A and TACC3.

## Data Availability

The data that support the findings of this study are available from the corresponding author upon reasonable request.
